# Motor Vehicle Safety – Has Technology and Legislation Made a Difference?

**DOI:** 10.1007/s40719-025-00282-6

**Published:** 2025-04-08

**Authors:** Matthew Kazaleh, Camille Meschia, Marie Crandall

**Affiliations:** 1 Department of Cardiac Surgery, University of Michigan, Ann Arbor, MI U.S.A.; 2 Department of Surgery, University of Florida Jacksonville, Jacksonville, FL U.S.A.; 3 Department of Surgery, Case Western Reserve University, Cleveland, OH 44106 U.S.A.

**Keywords:** MVC, Motor vehicle, Safety, Technology, Legislation

## Abstract

**Purpose of Review:**

Motor vehicle crashes are the leading cause of death among those less than 54 years old in the United States. From the genesis of automobile transportation, significant efforts to improve crash safety technology standards and traffic laws have been made. Despite these attempts, in 2021, the motor vehicle fatality rate increased by 10.5%, leading some to question the effectiveness of recent efforts. This critical review aims to discuss the history of motor vehicle technologic and legislative advancements, data investigating effectiveness, and opportunity for future improvement.

**Recent Findings:**

Collective transition to primary seatbelt laws, advancement of airbag design, implementation of driver assist technologies, as well as stricter alcohol legislation may offer response to the recent increase in motor vehicle fatalities.

**Summary:**

A coordinated effort for change from automobile manufacturers, drivers, law enforcement, and local/federal governments will be required to address the recent increase in motor vehicle fatalities.

## Introduction

Published journalist, Allison Glock once said, “Decisions can be like car accidents; sudden and full of consequences.” The National Safety Council estimates 282.4 million vehicles traveled 3.1 billion miles in the United States, in 2021 [1]. Millions of Americans rely on automobiles for transportation each day. While carrying ourselves and loved ones, we accept the risk of motor vehicle travel the moment we turn the ignition. Motor vehicle crashes are the leading cause of death among those less than 54 years old in the United States [2]. According to the National Highway Safety Administration, roughly 42,915 motor vehicle fatalities occurred in 2021 [3]. From the genesis of automobile transportation, engineers, manufacturers, and legislators have made valiant efforts to improve crash safety technology standards, roadway infrastructure, and traffic laws. Despite these attempts, in 2021, the motor vehicle fatality rate increased by 10.5%, leading some to question the effectiveness of recent efforts [3]. This critical review aims to discuss the history of motor vehicle technologic and legislative advancements, data investigating their effectiveness, and the opportunity for future improvement.

### Background and Significance

On January 29th, 1886, German engineer Carl Benz applied for a patent regarding his horseless carriage powered by a gasoline engine. Many would consider this to be the birth of the modern automobile that we have grown to know. It wasn’t until 1908, when American engineer Henry Ford, debuted his Model T that the automobile became widely available to the American public. By 1918, made possible by mass assembly lines, 5 million motor cars were registered in the United States, representing 85% of the world’s total automobiles. This marked the genesis of a problem, first made evident by road engineer Andrew Anderson, in 1917 – “How do we stop the usage of automobile transportation long enough for the transportation network to catch up?”.

**Fig. 1:**
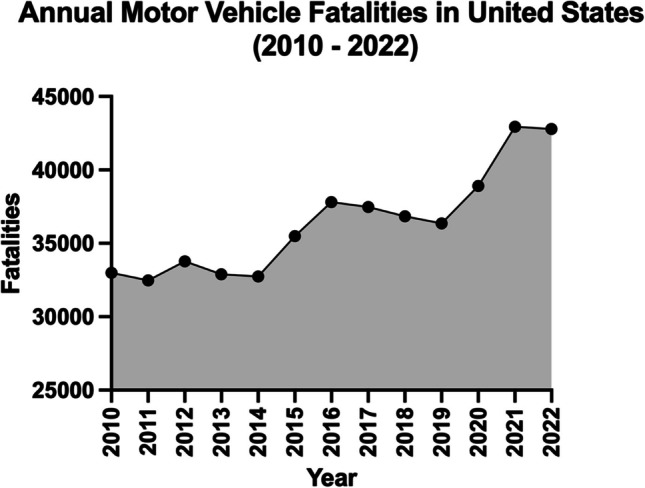
Annual motor vehicle fatalities in the United States from 2010 – 2022 (generated from averaged mortality data from National Highway Transportation Safety Administration and the United States Department of Transportation [1]) (created with Prism Graphpad Version 10, accessed 1 November 2023)

In 1913, 33.38 people died for every 10,000 vehicles on the road, whereas, in 2021, the death rate decreased to 1.66 deaths per 10,000 vehicles, representing a roughly 95% improvement [1]. Nevertheless, this comparison is fundamentally unequal, as these values do not represent 100 years of technological advancement. When looking at a comparison among more recent history, motor vehicle deaths increased by 22.9% from 2010 to 2022 **(**Fig. 1**)**. Why are motor vehicle fatalities continuing to increase, and what are automobile manufacturers and policy leaders doing to address this trend?

### Technology – Evolution of the Seatbelt:

The most influential safety innovation in the history of automobiles has been the seatbelt. Originally patented in 1885 by Edward Claghorn with the intention of ensuring the safety of taxi passengers in New York City, it wasn't until the 1930s that Dr. Claire Straith, a Detroit plastic surgeon, drew the attention of automobile manufacturers to the pressing need for this safety measure. Dr. Straith's motivation arose from the observation of a growing number of craniofacial injuries resulting from car accidents [4]. This pivotal moment sparked a movement toward developing occupant protective systems, with the seat restraint emerging as the simplest and most effective solution. By the 1950s, an increasing number of car manufacturers began incorporating seatbelts as standard equipment, and in 1968, the federal government mandated that all newly produced vehicles must be equipped with seatbelts. Lap and shoulder seatbelts play a vital role in reducing injuries to vehicle occupants by preventing direct contact with the vehicle's interior components or ejection during accidents. According to the Department of Transportation National Highway Traffic Safety Administration (NHTSA), seatbelts reduce the risk of death by 45% for passenger cars and 60% for light trucks, in addition to reducing the risk of serious injury by 50% [5, 6].

**Fig. 2:**
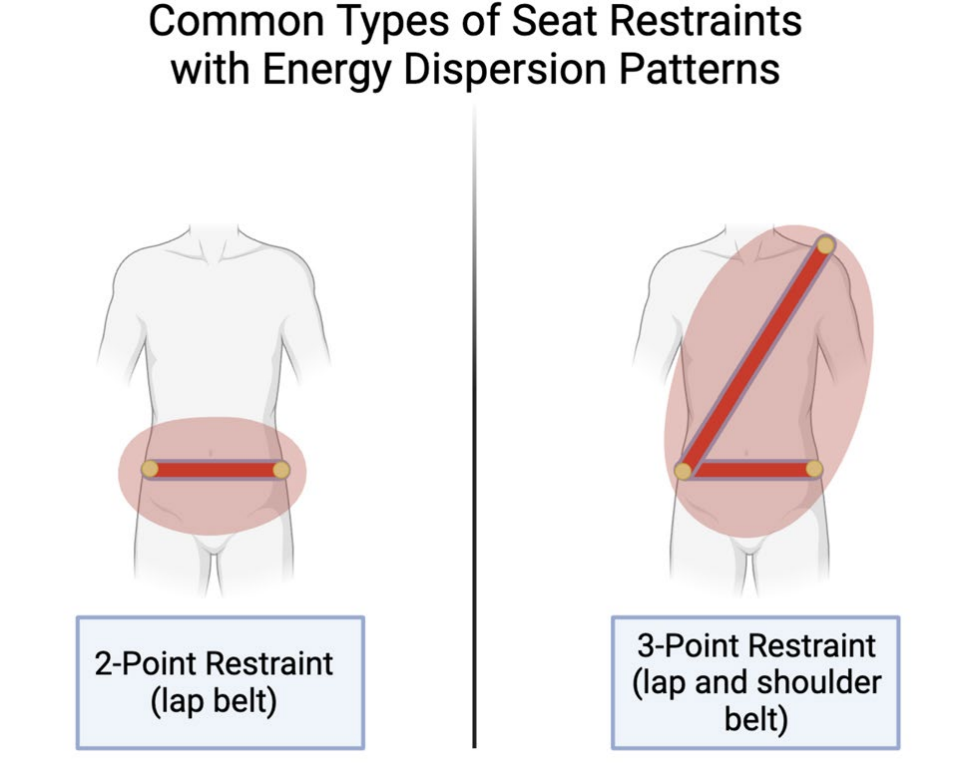
Depiction of 2-point and 3-point restraints with energy disbursement patterns (image generated via Biorender.com, accessed 1 November 2023)

However, not all seatbelts were created equal. The original design of restraints featured a single lap belt, often referred to as a two-point restraint. It wasn't until the late 1970s that the familiar lap and shoulder belt, known as the three-point restraint, became standard for front seat passengers (Fig. 2). This transition was partly influenced by research conducted by the NHTSA in June of 1999, which revealed that the use of lap belts alone in frontal car collisions substantially increased the risk of sustained abdominal injuries [7]. Conversely, the NHTSA found that lap/shoulder belts reduced abdominal injuries by 52% when compared to lap belts alone. They also reduced head injuries by 47% in comparison to lap belts alone.

These disparities can be attributed, in part, to the differing ways in which the two types of seatbelts disperse energy. Lap/shoulder belts distribute the energy resulting from occupant inertia across the shoulders, chest, and pelvis, whereas lap belts only focus energy on the pelvis. The physiologic implication of this physics is made evident in injury patterns. For example, the odds of a lumbar spine fracture in a pediatric passenger (age < 15 years) wearing a lap belt are 9 times higher when compared to lap/shoulder belts (95% CI 1.2–68.4) [8]. In September 2007, three-point seatbelts were required in both front and rear seats of all newly produced automobiles. Since their advent, minimal design changes have been made, impart due to simplicity and effectiveness.

The efficacy of seatbelts has been proven in various collision types, including head-on collisions. A retrospective analysis of 9,869 motor vehicle collisions involving 19,718 passengers from 1992 to 1997, from the Fatality Analysis Reporting System, at the University of New Mexico, explored this foundational principle further [9]. They found the use of lap/shoulder seatbelts to reduce mortality of front seat occupants in head-on collisions by 72% (OR = 0.28, 95% CI 0.25–0.31). This study also evaluated the mortality benefit of airbag deployment in conjunction with seat belts use and found a combined mortality reduction of 80% (OR = 0.18, 95% CI 0.13–0.25).

Similarly, Cummins et. al. sought to investigate the mortality benefit and reduction of injury severity with seatbelt usage [10]. They pooled 184,992 patients involved in motor vehicle collisions from the National Trauma Data Bank, from 1988 to 2004, and found a 51% mortality reduction (AOR = 0.49, CI 99% 0.45–0.52). When in conjunction with airbags, mortality reduced by 67% (AOR = 0.33, 99% CI 0.28–0.39). Injury severity was reported to exhibit similar patterns.

Interestingly, unbelted vehicle passengers can pose significant risk to other belted occupants. In 2004, MacLennan et al. established a 90% increased risk of injury of a belted occupant if an unbelted occupant was present in the vehicle at time of collision, as well as a 4.8-fold increased risk of mortality for the belted passenger (95% CI 3.3–7.0) [11]. In 2013, Bose et al. found that unbelted rear-seat passengers seated behind a belted front seat occupant increases fatality of the front seat passenger by 137%, even once controlling for driver age, sex, speed of vehicle, and airbag deployment (95% CI 95–189) [12].

Abundant data underscores the life-saving advantages of seatbelts for all occupants in motor vehicle collisions. This assertion has been irrefutably supported with data over the past 30 years. However, the extent of this life-saving advantage hinges on whether the occupants make use of this crucial technology. Thus, the opportunity to improve motor vehicle crash injury and mortality stems from optimizing legislation to encourage compliance.

### Legislation – Seatbelts

Seatbelts have proven effective in mitigating the risk of fatal injuries in motor vehicle accidents. Although mandatory installation was established in 1968, their usage was left to the discretion of vehicle occupants until recently. In 2000, the Transportation Research Board reported that approximately 71% of front seat passengers utilized safety belts [13]. By 2010, this figure had risen to 85%, partially attributed to legislative measures. These legislative efforts took the form of both primary and secondary seatbelt laws. Primary seatbelt laws empower law enforcement officers to issue citations to occupants for failing to wear seatbelts without the need for other traffic violations [14]. The inception of the first primary seatbelt law occurred in New York State in 1984, and by 2010, as many as 30 states had enacted primary seatbelt laws [14].

Secondary laws allow citations for seatbelt noncompliance only when law enforcement has stopped a vehicle for a different reason. Enforcing secondary laws poses more challenges, in part because of the perception that secondary violations may not be as legally enforceable in court [15]. The presence of both primary and secondary seatbelt laws has been associated with a 3% increase in compliance; however, primary laws have been found even more efficacious [16]. Shults et al. found in 2004 that states transitioning from secondary to primary enforcement experienced a 14% rise in seatbelt usage and an 8% reduction in fatal injuries [17]. Similar findings have been reported in other studies, indicating an estimated 7% decrease in driver fatalities in states transitioning from secondary to primary seatbelt laws [18]. These studies show that the implementation of seatbelt laws has effectively fostered compliance with seatbelt use. In 2006, Beck et al. analyzed data from the Behavioral Risk Factor Surveillance System, revealing that 86% of drivers in states with primary enforcement used seatbelts, compared to 75.9% in states with secondary enforcement [19]. Given these findings, we propose that transitioning to primary seatbelt enforcement for all states may result in a reduction in automotive collision mortality [20].

According to the NHTSA, as of 2019, 34 states have primary seatbelt laws, while 15 still have secondary laws [21]. New Hampshire remains the only state currently without seatbelt legislation. This inequality cannot be overlooked, especially as we investigate the recent increase in motor vehicle fatalities.

### Technology – Airbags

Similar to the advent of seatbelts, the development of airbags represents a significant safety technological advancement which has profoundly reduced mortality associated with automobile collisions. Invention of the airbag is credited to United States Naval industrial engineer John Hetrick, in 1953, as he sought to develop a “safety cushion assembly” for automobiles [22]. Referred to as a “passive restraint system”, his initial design consisted of inflation of a bladder with compressed air upon vehicle impact. These inflatable cushions prevent vehicle occupants from striking automobile interior components, as well as preventing injury from forcible flexion during periods of expedient deceleration. Although foundational in concept, his design would undergo several significant changes until official implementation into production vehicles by the early 1970s. This implementation was made possible through numerous design improvements by mechanical engineer, Allen Breed, in the 1960s [23, 24]. Breed is credited with creation of electromechanical crash detection sensors which would ignite an explosion of sodium azide, filling airbags in under 30 ms [24, 25]. It was this speedy inflation time which made possible the efficacy of airbag deployment during collisions. As early as 1976, studies identified mortality benefits of airbags during automotive collisions [26].

The two main types of airbags in modern production vehicles are front and side airbags. In 1998, the federal government mandated all new production vehicles be equipped with front airbags via the Transportation Equity Act [26]. At the time, foundational studies from Barry et. al employed multivariate conditional logistic regression models to evaluate the mortality benefit of airbag use while accounting for confounding variables such as occupant age, sex, seatbelt use, and location within the vehicle during frontal collisions [26]. The group proved a mortality benefit with the use of airbags, although not as profound as initially anticipated. The mortality benefit differed among age and sex groups, with the largest benefit in males and young adults who were wearing seatbelts at time of collision. This study drew key limitations of the current airbag technology of the time, which spurred the modern age of airbag technology research and development.

Side airbags play a crucial role in preventing injuries, typically addressing head and upper torso trauma, especially in perpendicular collisions [27, 28]. There are two fundamental types of side airbags: side-impact airbags, commonly situated in rear doors to shield the chest and abdomen, and curtain airbags, which deploy from above the door to safeguard occupants' heads from potential collisions with windows or external materials.

In 2006, McCartt et al. conducted an assessment on the effectiveness of deploying side airbags in diminishing driver fatalities resulting from side collisions involving both cars and SUVs. Their findings revealed a 37% decrease in the risk of driver mortality when head-protecting airbags were employed and a 26% reduction with torso-only protecting airbags in cars [29]. The mortality risk reduction was slightly more pronounced in SUVs, reaching 52% with head-protecting side airbags and 30% with torso-only airbags. Recent literature strongly supports the efficacy of contemporary airbags in reducing injuries and fatalities among vehicle occupants. In 2015, Kahane et al. reported a 29% and 32% reduction in fatalities for front seat passengers and drivers, respectively, through the deployment of airbags during collisions [26–28]. According to the NHTSA, the combination of seatbelt usage and airbag deployment in frontal collisions leads to a 61% reduction in mortality [27]. The evidence remains clear, much like seatbelt use, airbags save lives.

### Legislation – Airbags

Unlike seatbelts, which require occupant compliance with or without federal and state laws, the mortality benefit of airbags is inquired without action from drivers and occupants. This is why the term “passive restraint system” has been used to describe airbags. Thus, legislation mandating airbag implementation has had immediate and profound effects on motor vehicle collision mortality. As previously discussed, the airbag mandate in 1998 has proven mortality benefit. According to the NHTSA, as of 2017, an estimated 50,457 lives have been saved by frontal airbags [30]. While there isn't a universal mandate for side airbags, federal regulations do require specific standards for the head and torso safety of all vehicle occupants. This regulatory gray area has led to various iterations of airbag designs and represents an area of universal improvement in motor vehicle safety. In general, airbag legislation has ensured performance standards through rigorous testing and certification.

In the United States, the NHTSA is the agency responsible for establishing and enforcing safety standards in production vehicles through the Federal Motor Vehicle Safety Standard (FMVSS) requirement. This regulation monitors airbag technology, ensuring engineers account for factors such as differences in occupant weight, size, position within vehicles, and vector of collision. Currently, airbag safety remains a critical aspect of motor vehicle development, as automotive engineers address the evolving needs of vehicle safety. Perhaps this evolution is occurring too rapidly, with the progress of automobile travel and accessibility potentially surpassing the development of safety mechanisms within the last 10 years.

### Technology – Advanced Driver Assist (ADA) Technologies

Majority of automobile collisions result from human error, highlighting the pressing need for innovative solutions to enhance road safety. In response to this challenge, automobile engineers have sought to develop and implement a multitude of advanced driver assist (ADA) technologies. These technologies encompass a wide range of features including rear-viewing cameras, forward collision prevention, lane-keeping assistance, and blind zone detection. These cutting-edge advancements aim to mitigate the impact of human error on collisions, ultimately creating a safer driving ecosystem for all road users. ADAs assist drivers by integrating steering, vehicle speed, and environmental hazard data for suggested corrections. Although extremely advanced, the classification of ADAs require user engagement. This is an important distinction from completely autonomous motor vehicles. It is estimated that more than 50% of all registered vehicles in the United States will have a form of ADA by 2026 [31]. According to the NHSTA, recent studies predict the use of advanced driver assist technologies could prevent 1.69 million injuries and 20,841 deaths annually [31–33].

The promising initial data supporting ADA use has also spurred legislative changes. In 2021, the Bipartisan Infrastructure Law (BIL) highlighted the importance of ADAs by requiring all new vehicles to be equipped with some form of advanced driving assistance. The BIL also dedicated significant funding ($108 billion) towards automated driving and advanced driver assistance technology research, representing the largest federal investment in infrastructure and transportation in US history [34, 35]. Although further research efforts investigating the efficacy of specific ADAs is required, we argue the implementation of additional ADAs could improve motor vehicle collision injuries and fatalities.

### Technology – Breakaway Steering Column

Additional technology with potential for injury reduction are breakaway/collapsible steering columns. At the point of impact during a head-on collision, the steering wheel represents a significant danger to occupants. As momentum forces drivers into a newly static vehicle interior cervical airway damage, maxillofacial trauma, blunt cardiac injury, and thoracic aortic dissection represent a few of the many traumatic injuries which can result from contact with fixed steering columns, even despite the presence of airbag deployment [36–39]. In fact, this association is so pronounced that steering wheel deformity during motor vehicle collisions has been identified as an independent predictor of serious thoracic injury of drivers and front seat occupants (OR = 1.28 for each 5-cm increase in steering wheel deformity, 95% CI 1.04 – 1.59) [40]. To combat this, automotive engineers have developed steering columns which detach and internally telescope during collision, absorbing and preventing impact of the driver’s head and thorax [41]. Although in its infancy, in 2022 the NHTSA recommended collapsible steering columns for means of frontal impact safety standards in all new production vehicles given promising data [42].

### Technology – Safety Glass

Injury from windshield shattering also imparts a significant threat to vehicle occupants. Lacerations from large mobile glass fragments, as well as occupant ejection are all results of the loss of windshield integrity during motor vehicle collisions [43]. Historical data (1989) suggests occupants who are ejected from a vehicle are up to 8-times more likely to be killed compared to those not ejected [44]. Vehicle ejection prevention is associated with a 70% reduction in mortality [45, 46]. In addition to seatbelts, engineers have addressed this problem through design and implementation of laminated and tempered automotive glass. Tempered glass, produced by molding and rapidly cooling sheets of glass to impart increased hardness, shatters into many small pieces when broken [47–49]. This results in loss of windshield integrity, essentially opening the vehicle cabin for objects to enter or for passengers to be ejected. Conversely, laminated glass is produced with a plastic interlay, resulting in maintained integrity when the windshield shatters [47–49]. By coating glass with this plastic interlay, laminated glass can prevent occupant ejection, ultimately reducing mortality. Although laminated glass windshields were first introduced by Henry Ford, in 1927, traditionally weak safety standards of automotive glass have led to delays in complete implementation [49]. Modern day production vehicles employ mostly tempered glass construct. Manguso et. al. reassessed the trend of ejection in modern day vehicles and found a 55.7% reduction in ejection rate from 2002 to 2012 (p < 0.01), providing a hopeful future for automotive safety innovation [50]. Current ongoing research regarding optimal windshield glass design could further reduce mortality rates [51].

### Alcohol-Impaired Driving

In 2020, 30% of all motor vehicle related crash deaths were a result of alcohol-impaired driving (AID), accounting for a $123.3 billion burden on the healthcare system [52, 53]. This represented an increase of 14.3% compared to the AID crash related mortality in 2019 [52, 53]. Of those fatalities, 62% were intoxicated themselves, while the remaining 38% were passengers or drivers of another vehicle [52, 53]. Regardless of the robust and cutting-edge safety features on modern day vehicles previously discussed, physical impairment of drivers results in a significant portion of motor vehicle fatalities each year. However, unlike the safety concerns of early motor vehicle development which were solved with technological advancement, AID poses a systematic problem requiring a much different approach. We maintain that legislation acts as the only means of addressing AID associated mortalities, and optimization of this legislation should be the first approach to addressing this increase in mortality.

Arguably the most historic and commonly used recreational drug, the effects of ethanol (EtOH) on the human body have been extensively studied. Categorized as a central nervous system depressant, EtOH consumption in certain quantities can result in feelings of happiness, euphoria, and decreased anxiety [54, 55]. The liver is responsible for the majority of EtOH metabolism into ketones, via enzyme Alcohol Dehydrogenase, which occurs at a rate of 0.015 g/100 mL/hour [56]. This number is subject to alterations based upon a variety of physiologic differences. Issues arise when the consumption of EtOH at a rate faster than detoxification occurs [57, 58]. As EtOH concentration builds in circulation, allosteric GABA A receptor modulation results in cognitive, sensory, and motor impairment [59]. According to the Cleveland Clinic, decreased environmental awareness and impaired judgment can occur with BAC of 0.05% [60]. BAC of 0.08% is associated with reduced muscle coordination and cognitive reasoning [60]. BAC of 0.10% results in reduced reaction time, slurring speech, and deterioration of fine motor movements [60]. Of note, BAC sampling is only accurate within 6–12 h following the time of last alcoholic consumption, due to metabolism [61]. Currently, AID is defined as the operation of a motor vehicle while under the influence of alcohol with a blood alcohol content (BAC) of greater than or equal to 0.08%.

The first law criminalizing the operation of a motor vehicle while intoxicated occurred in New York, in 1910 [62]. However, specifics of the definition of “intoxication” were not defined at that time. In 1933, following the prohibition era, awareness of AID increased. With the advent of the “breathalyzer” in 1954, by Robert Borkenstein, enforcement of AID laws became a reality [63]. Prior to 1980, states determined the legal limit to be BAC < 0.10%. This would later be adjusted to 0.08% by Utah in 1983, followed by several states in the 1990s [64]. Public awareness of AID heightened in the 1970s with federally funded campaigns such as Mothers Against Drunk Driving (MADD) [65]. The Transportation Equity Act (2000) included federal funding to those states who adopted and enforced legal BAC < 0.08% [66]. By 2004, all 50 states had adopted the 0.08% BAC limit. Fell et. al. explored the potential benefit of this change [67]. Through review of fourteen independent studies in the US, they found a 5–16% reduction in AID related collisions, injuries, and fatalities with the change from BAC 0.10 to 0.08. They also explored the potential benefit of further reduction to 0.05. They found the relative risk of fatal AID to be 4 to 10 times greater for individuals with BAC between 0.05 and 0.07. This further makes evident that critical impairment of driving associated with cognitive and motor skills can occur with a BAC of > 0.05.

Lowering the legal BAC limit poses a potential approach to the reduction of AID associated injuries and mortality, as made evident by the effects of recent legislation changes. In May of 2013, the NTSB proposed that states lower the BAG limit from 0.08% to 0.05% [68, 69]. Although not approved by most states, in 2017, Utah made this change. Following this legislation, the state of Utah experienced a fatality rate reduction of 18.3%, providing evidence of the potential efficacy of this change [68**··**]. Surveys were conducted and found that 22.1% of drivers indicated they have altered their drinking behaviors due to these recent law changes.

Additional legislation efforts made to reduce AID associated collisions and fatalities include the implementation of ignition locking mechanisms. These locking mechanisms consist of a breathalyzer interlinked with the ignition of vehicles. With BAC over a certain limit, the ignition will not start the vehicle, preventing the intoxicated individual from driving. Currently, 30 states have mandatory alcohol ignition interlock law for all drivers who have been convicted of driving while under the influence of alcohol [70]. Kaufman et. al. employed data from the NHTSA from 2004 to 2013 and found that the absolute reduction of AID fatality rates for states with interlock laws was 0.8 (95% CI 0.1—1.5) deaths per 100,000 [71]. They concluded that requiring ignition interlocks for all AID convictions was associated with 15% fewer AID related fatalities. Recent data suggests states who implemented some iteration of these laws saw a reduction in AID. Teoh et. al. evaluated different types of state legislation from 2001 to 2019. They found laws for anyone who had been convicted of AID saw 26% fewer drivers with BAC > 0.08 involved in fatal crashes compared to states with no laws [72, 73]. States with requirements for interlock systems in drivers who had repeat offenses saw a 9% reduction in AID. The recidivism rate of AID has been reported significantly lower with the use of ignition interlocking systems (15—69% less likely when compared to control) [74, 75].

## Conclusion

Through the advent of the 2- and 3-point seat restraints, front and side curtain airbags, advanced driver assist technologies, breakaway steering columns, shatter resistant glass, and alcohol impaired driving laws mortality associated with motor vehicle travel has dropped precipitously. Through technologic advancement, there has been a general downtrend of motor vehicle fatalities (95% overall reduction in percentage of mortalities since 1913). This trend continued throughout modern vehicle design, as made evident in collision mortality rates of the early 1970s compared to today (26.9% in 1972 to 11.1% per 100,00 people in 2014) [1]. Despite this, current efforts to improve the safety of motor vehicle travel are needed given the recent surge in motor vehicle fatalities within the last 10 years. Recent literature suggests collective transition from secondary to primary seatbelt laws, continued advancement and implementation of airbag design, driver assist technologies, as well as stricter alcohol impaired driving legislation may offer response to the recent increase in motor vehicle fatalities. Through a coordinated and collective effort from automobile manufacturers, drivers, law enforcement, and local/federal governments we can correct the trajectory of motor vehicle safety, just as we have done in the past.

## Data Availability

No original date was used for the creation of this work.
